# Anterior Segment Tomography with the Cirrus Optical Coherence Tomography

**DOI:** 10.1155/2012/806989

**Published:** 2012-01-24

**Authors:** Eduardo B. Rodrigues, Margara Johanson, Fernando M. Penha

**Affiliations:** ^1^Department of Ophthalmology, Vision Institute, UNIFESP, Sao Paulo, Brazil; ^2^Instituto de Olhos de Florianópolis, Rua Presidente Coutinho 579, Conjunto 501, 88000 Florianópolis, SC, Brazil

## Abstract

Optical coherence tomography (OCT) is an optical acquisition method to examine biological tissues. In recent years, OCT has become an important imaging technology used in diagnosing and following macular pathologies. Further development enabled application of optical coherence tomography in evaluation of the integrity of the nerve fiber layer, optic nerve cupping, anterior chamber angle, or corneal topography. In this manuscript we overview the use of OCT in the clinical practice to enable corneal, iris, ciliary body, and angle evaluation and diagnostics.

## 1. Introduction

Optical coherence tomography (OCT) systems use low-coherence, near-infrared light to provide detailed images of anterior segment structures at resolutions exceeding other systems like ultrasound biomicroscopy or conventional ultrasound [1, 2]. The first use of OCT for anterior segment imaging has been reported by Izatt and coworkers, who developed a slit-lamp-mounted, 830 nm time-domain device [3]. The initial equipment allowed direct in vivo measurements of corneal thickness and surface profile, anterior chamber depth and angle, and iris thickness and surface profile. Since then, significant technology progress has been made which ultimately enabled establishment of the current time-domain Visante OCT as well as the spectral-domain OCT device. The Cirrus spectral-domain equipment (Carl Zeiss Meditec, Dublin, CA, USA) is an around 5 micra high-definition (HD) 840 nm spectral-domain OCT instrument primarily designed for retinal imaging.

The purpose of this paper is to present the various applications of the HD-OCT Cirrus device in the clinical practice to enable corneal, iris, ciliary body, and angle evaluation, for instance, examination of even fine structures like Descemet's membrane, the trabecular meshwork, and Schwalbe's line.

## 2. Acquiring Anterior Segment Scans with the Cirrus

The Anterior Segment Cube 512 × 128 mode generates a volume of data through a 4-millimeter square grid by acquiring a series of 128 horizontal scan lines each composed of 512 A-scans. The mode acquires a pair of high-definition scans through the center of the cube in the vertical and horizontal directions that are composed of 1024 A-scans each. The Anterior Segment Cube 512 × 128 can be used for measuring the central corneal thickness and create a 3-D image of the data.

The Anterior Segment 5-Line Raster scans through 5 parallel lines of equal length that can be used to view high-resolution images of the anterior chamber angle and cornea. The line length is fixed at 3 mm, while the rotation and spacing are adjustable. Each line is composed of 4096 A-scans, and by default, the lines are horizontal and separated by 250 *μ*m, so that the 5 lines together cover 1 mm width.

## 3. Corneal Evaluation

### 3.1. Indications and Limitations

**Figure 1:**
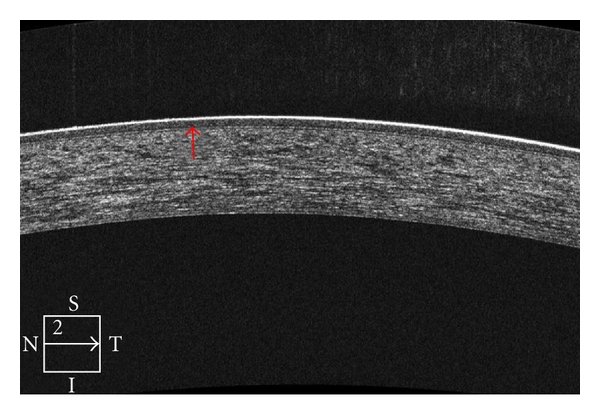
High-definition OCT of the cornea enables localization of the interface between the corneal stroma and epithelial/Bowman's layer.

**Figure 2:**
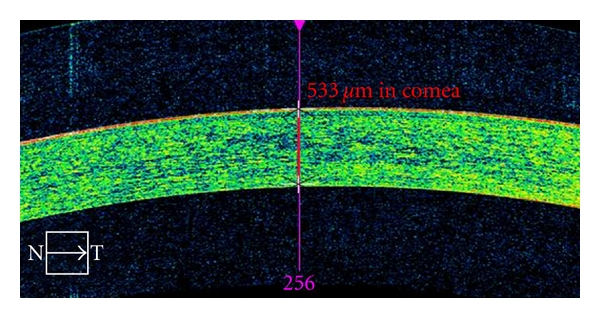
Anterior Segment Cube 512 × 128 imaging of the cornea enables pachymetry measurements.

The HD-OCT device has been recently used as a noninvasive corneal imaging modality that was capable of in vivo differentiation of both corneal layers and demonstration of pathologic abnormalities in the cornea (Figure 1). Corneal indications for the Cirrus OCT can be divided into pachymetry analysis, keratectomy, refractive surgery, structural, and contact lens abnormalities. For pachymetry, the Anterior Segment Cube 512 × 128 enables identification of the central corneal section, which facilitates positioning for central corneal thickness measurements. Images are framed in blue and pink in order to demonstrate the vertical and horizontal line (Figure 2). No specific pachymetry map on the Cirrus is yet provided. Further studies should be performed to compare the validity of the Cirrus pachymetry with other devices, for example, Pentacam.

For refractive surgery corneal Cirrus OCT enables identification of the postoperative flap and unexpected changes as epithelial ingrowth. The thickness of eventual epithelial hyperplasia can be viewed by the Cirrus OCT. Postoperatively, OCT enables visualization of corneal flaps, as well as the integrity of the corneal layers.

For keratectomy the HD-OCT also allows various changes such as attachment of the implanted posterior Descemet in DMEK procedure or even wrinkle or fold within the DSEK. The OCT images also enable better clinical decision to conduct more accurate planning of treatments for corneal opacities.

**Figure 3:**
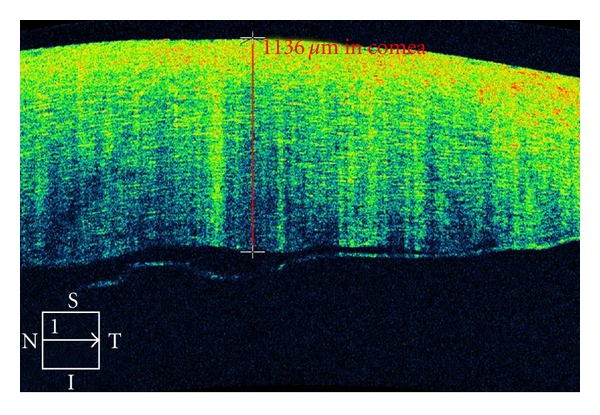
Cirrus OCT of a 79-year-old patient who underwent cataract surgery. She developed postoperative corneal edema detected and monitored by the Cirrus OCT, characterized by diffuse hyperreflectivity interspacing lacunae of hyporeflectivity on corneal OCT scan. In addition, interruption of the endothelial layer can be documented.

Structural anatomies of the cornea like keratin precipitates secondary to uveitis can be monitored with the Cirrus OCT. Cases of intrastromal corneal foreign body can be viewed by the Cirrus. OCT accurately maps corneal thickness in clear and opacified corneas, allowing the examiner to precisely map the depth of corneal opacities, the degree of epithelial hyperplasia, and the thickness of the cornea, as well as corneal edema and thickening, scleral melts, corneal degenerations, and scars as well as corneal dystrophies [4] (Figure 3). Cirrus OCT provides clear delineation of corneal anatomic features and pathologic corneal deposits in most cases. The characteristics and depth of these deposits are illustrated and can be localized to specific layers of the cornea. When available, there has been significant correlation between OCT images and histopathologic features, providing a noninvasive confirmation of the clinical diagnosis.

Contact lens distance to the corneal epithelial can be measured with the Cirrus OCT. It enables to distinguish cases of thinner tear film thickness, which may be a sign of tight contact lens adaptation. Some of the limitations of the Cirrus in corneal analysis include not enough resolution for endothelial cell distinction, less precise pachymetry in comparison to the Pentacam, and little amount of publications on the topic in the medical literature.

### 3.2. Selected Clinical Cases

**Case 1**

**Figure 4:**
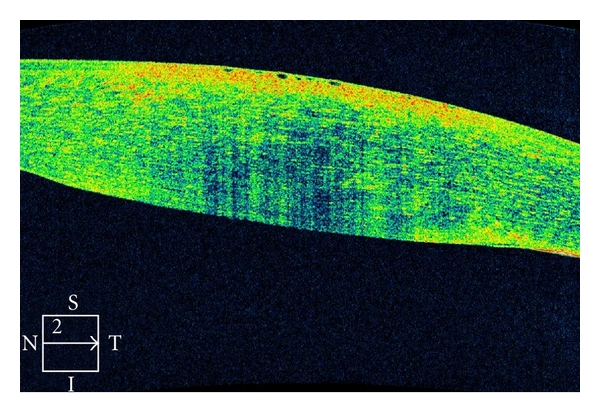
Corneal imaging with the Cirrus OCT of an 86-year-old female patient who underwent lens phacoemulsification for therapy of dense nigra cataract. She developed focal microbullous keratopathy. OCT shows both the remarkable corneal edema, as well as interruption of endothelium layer. The patient was treated with topical steroids and experience improvement 4 weeks later.

An 86-year-old female underwent phacoemulsification surgery for therapy of cataracta rubra, advanced nuclear sclerosis. The surgery lasted 28 minutes while ultrasound time 3.02 minutes.  Figure 4 shows microbullous keratopathy after phacoemulsification with dense corneal edema and interruption of endothelium-Descemet's membrane layer.

**Case 2**

A 77-year-old patient presents with nuclear lens cataract. Preoperative evaluation included specular corneal microscopy, fundus evaluation, and pachymetry. Figure 2 demonstrates the utility of the CIRRUS for measurement of the total central corneal thickness of 533 micra. Recent research demonstrated accordance of these outcomes with the pachymetry data [5].

## 4. Anterior Chamber Angle Evaluation

### 4.1. Indications, Limitations, and Comparison with the Visante OCT

The Visante anterior segment OCT system (Carl Zeiss Meditec, Dublin, CA, USA) is a time-domain 1310 nm operating device that supports several modes, including high-resolution cornea, corneal pachymetry, and anterior segment at resolution of 18 *μ*m axially by 60 *μ*m laterally. The Visante anterior segment OCT (AS-OCT) has been proposed as a diagnostic tool to evaluate gonioscopic angle closure in patients; however, it may overestimate the frequency of closed angles compared to gonioscopy [6]. This discrepancy in findings may occur because on AS-OCT images, it is not possible to determine the location of the trabecular meshwork, and the presence of any contact between the iris and the angle wall anterior to the scleral spur is graded as angle closure. However, if this apposition did not reach the level of the posterior trabecular meshwork, the quadrant would be considered open on gonioscopy. The inability to detect the scleral spur may limit the accuracy and usefulness of angle imaging, methods as AS-OCT.  Table 1 describes some instrument properties and differences among Visante, Pentacam, and Cirrus OCT.

**Figure 5:**
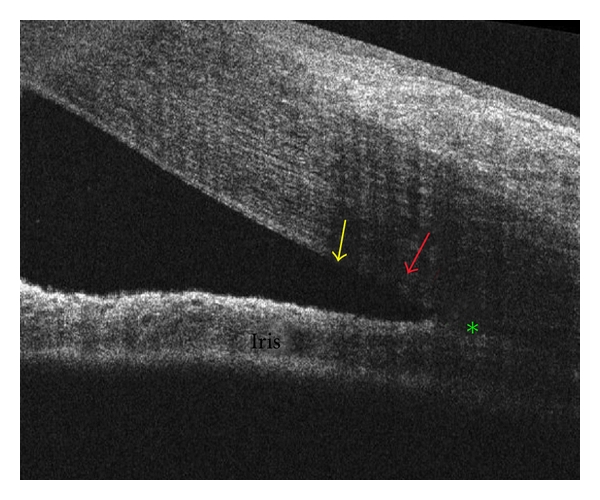
The Cirrus HD-OCT may enable identification of Schwalbe's line (yellow arrow), scleral spur (green asterisk), and trabecular meshwork (red arrow).

**Figure 6:**
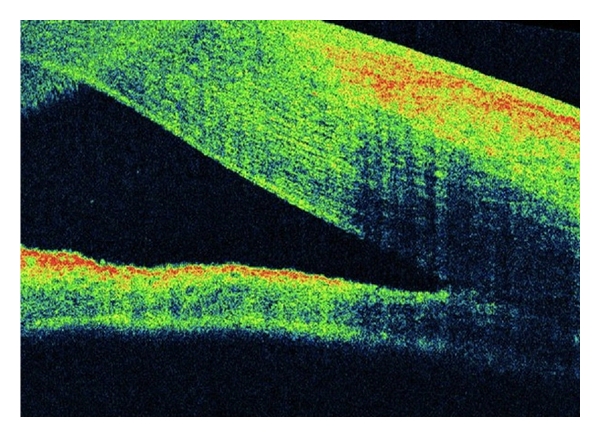
A 45-year-old female underwent vitrectomy plus silicone oil for retinal detachment repair. She presented with postoperative corneal edema that impaired angle examination. Cirrus OCT disclosed open anterior chamber angle.

HD-OCT applies a scanning rate 50 to 60 times faster than time-domain OCT devices and with an axial resolution of 3 to 5 *μ*m. Cirrus HD-OCT makes images of the anterior chamber angle with higher resolution than does AS-OCT devices. With the Cirrus OCT, details of the anterior chamber angle such as the scleral spur can be viewed. The Cirrus OCT may enable identification of Schwalbe's line, scleral spur, and trabecular meshwork (Figure 5). It also assists in the diagnosis of narrow angle or anterior iris synechia (Figure 6). When analyzing the usefulness of HD-OCT in angle examination, the rate of angle closure diagnosis was lower using HD-OCT, with some eyes graded as open on HD-OCT but closed on gonioscopy. Wong et al. assessed the ability of HD-OCT with a 60-diopter aspheric lens mounted over the imaging aperture to image the anterior chamber angle [7]. Cross-sectional HD-OCT enabled visualization of the scleral spur in 71 of 90 quadrants (78.9%) and the termination of the Descemet membrane (Schwalbe's line) in 84 of 90 quadrants (93.3%). The authors concluded that the adapted HD-OCT allowed magnified views of the anterior chamber angle and provided visualization of Schwalbe's line and trabecular meshwork in most eyes. The newest version of the Cirrus software 4.0 presents improvements that appropriate anterior segment examination without the need of any lens. One recent study claimed spectral domain OCTs as the better means to identify Schwalbe's line in comparison with other devices [8]. Future studies should promote improvements in the application of HD-OCT in angle imaging for instance analysis with the 3-dimensional image of the angle.

### 4.2. Selected Clinical Case

**Case 1**

A 45-year-old female underwent vitrectomy plus silicone oil for retinal detachment repair. She presented with postoperative corneal edema and increase in intraocular pressure. The corneal edema impaired angle examination. OCT disclosed open anterior chamber angle (Figure 6).

**Case 2**

**Figure 7:**
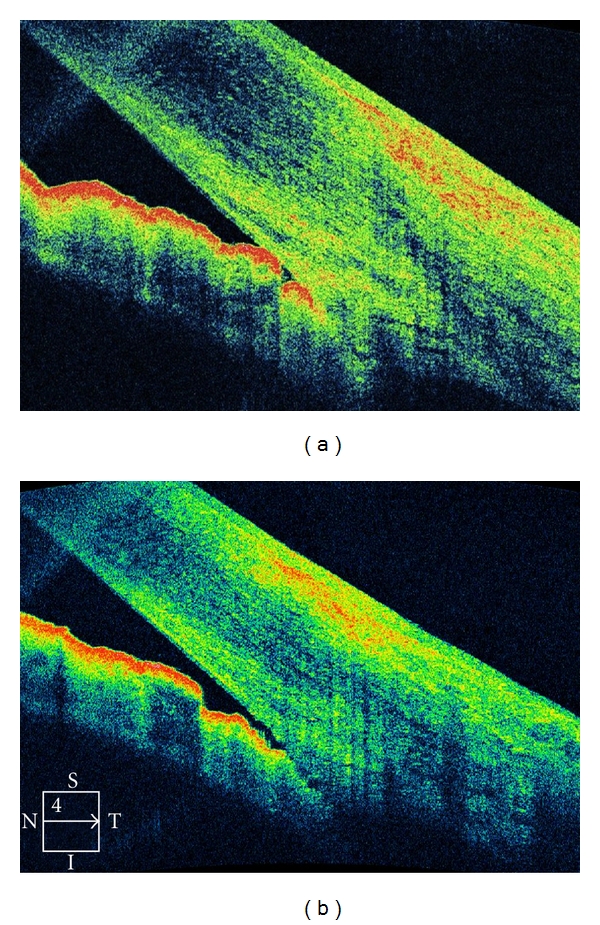
A 49-year-old female presents with a 1-week history of pain and visual loss OS. Ophthalmic examination was unremarkable, except for angle alterations and intraocular pressure of 57 mmHg OS. There were OS areas alternating very narrow and close anterior chamber angle; OD the angle was narrow. (a) OCT examination confirmed the anterior chamber angle morphology (b) The patient underwent topical and systemic antihypertensive ocular therapy. Two days later, the intraocular pressure was 16 mmHg OS, the angle was open, confirmed by OCT exam.

A 49-year-old female presents with a 1-week history of pain and visual loss OS. Her corrected visual acuity was 20/80 OS and 20/20 OD. Ophthalmic examination was unremarkable, except for angle alterations and intraocular pressure of 57 mmHg OS. There were OS areas alternating very narrow and close anterior chamber angle; OD the angle was narrow. OCT examination confirmed the anterior chamber angle morphology (Figure 7(a)). The patient underwent topical and systemic antihypertensive ocular therapy. Two days later, the intraocular pressure was 16 mmHg OS, the angle was open, confirmed by OCT exam (Figure 7(b)).

## 5. Final Remarks

Cirrus OCT provides relevant clinical data in regard to anterior chamber and corneal diagnosis. It enables detailed high-resolution corneal morphology analysis. It also allows anterior-chamber angle examination. OCT is a novel device to perform in vivo optical biopsies and a promising research and clinical tool for the evaluation of corneal pathologic features in a noninvasive manner. The future use of this novel technology should develop and increasingly is becoming important equipment in the clinical and surgical management of corneal, anterior chamber angle, and iridociliary diseases.

## Figures and Tables

**Table 1:** Some properties and differences among Visante, Pentacam, and Cirrus OCT are described in detail. Courtesy of Zeiss.

(I) Visante anterior segment OCT
(a) Anterior segment
(i) 6 mm depth by 16 mm width 256 A-Scan per B-Scan
(ii) 3 mm depth by 10 mm width 512 A-Scan per B-Scan
(b) Wavelength 1310 nm (infrared, nonvisible light)
(II) Cirrus HD-OCT
(a) Anterior segment
(i) Cube: 4 × 4 mm, 512 A-Scan
(ii) 5-Line Raster: 3 mm, 4096 A-Scans
(b) Posterior segment
(i) Cube: 6 × 6 mm, 512 A-Scan
(ii) 5-Line Raster: 9 mm, 4096 A-Scans
(c) Wavelength 840 nm
(III) Pentacam
(a) Rotating Scheimpflug Imaging System
(b) Wavelength 475 nm, blue LED (Info: Ziemer Galilei wavelength 470 nm)

## References

[1] Doors M, Berendschot TTJM, de Brabander J, Webers CAB, Nuijts RMMA (2010). Value of optical coherence tomography for anterior segment surgery. *Journal of Cataract and Refractive Surgery*.

[2] Simpson T, Fonn D (2008). Optical coherence tomography of the anterior segment. *Ocular Surface*.

[3] Izatt JA, Hee MR, Swanson EA (1994). Micrometer-scale resolution imaging of the anterior eye in vivo with optical coherence tomography. *Archives of Ophthalmology*.

[4] Ramos JLB, Li Y, Huang D (2009). Clinical and research applications of anterior segment optical coherence tomography—A review. *Clinical and Experimental Ophthalmology*.

[5] Kim HY, Budenz DL, Lee PS, Feuer WJ, Barton K (2008). Comparison of central corneal thickness using anterior segment optical coherence tomography vs. ultrasound pachymetry. *American Journal of Ophthalmology*.

[6] Sakata LM, Lavanya R, Friedman DS (2008). Comparison of gonioscopy and anterior-segment ocular coherence tomography in detecting angle closure in different quadrants of the anterior chamber angle. *Ophthalmology*.

[7] Wong HT, Lim MC, Sakata LM (2009). High-definition optical coherence tomography imaging of the iridocorneal angle of the eye. *Archives of Ophthalmology*.

[8] Jing T, Marziliano P, Wong HT Automatic detection of Schwalbe’s line in the anterior chamber angle of the eye using HD-OCT images.

